# Early career scientists converse on the future of soft robotics

**DOI:** 10.3389/frobt.2023.1129827

**Published:** 2023-02-22

**Authors:** Falk J. Tauber, Viacheslav Slesarenko

**Affiliations:** ^1^ Cluster of Excellence livMatS, FIT—Freiburg Center for Interactive Materials and Bioinspired Technologies, University of Freiburg, Freiburg im Breisgau, Germany; ^2^ Plant Biomechanics Group (PBG) Freiburg, Botanic Garden of the University of Freiburg, Freiburg im Breisgau, Germany

**Keywords:** soft robotics, bio-inspiration, energy autonomy, adaptivity, sustainability, perspective

## Abstract

During the recent decade, we have witnessed an extraordinary flourishing of soft robotics. Rekindled interest in soft robots is partially associated with the advances in manufacturing techniques that enable the fabrication of sophisticated multi-material robotic bodies with dimensions ranging across multiple length scales. In recent manuscripts, a reader might find peculiar-looking soft robots capable of grasping, walking, or swimming. However, the growth in publication numbers does not always reflect the real progress in the field since many manuscripts employ very similar ideas and just tweak soft body geometries. Therefore, we unreservedly agree with the sentiment that future research must move beyond “soft for soft’s sake.” Soft robotics is an undoubtedly fascinating field, but it requires a critical assessment of the limitations and challenges, enabling us to spotlight the areas and directions where soft robots will have the best leverage over their traditional counterparts. In this perspective paper, we discuss the current state of robotic research related to such important aspects as energy autonomy, electronic-free logic, and sustainability. The goal is to critically look at perspectives of soft robotics from two opposite points of view provided by early career researchers and highlight the most promising future direction, that is, in our opinion, the employment of soft robotic technologies for soft bio-inspired artificial organs.

## 1 Introduction

**FIGURE 1:**
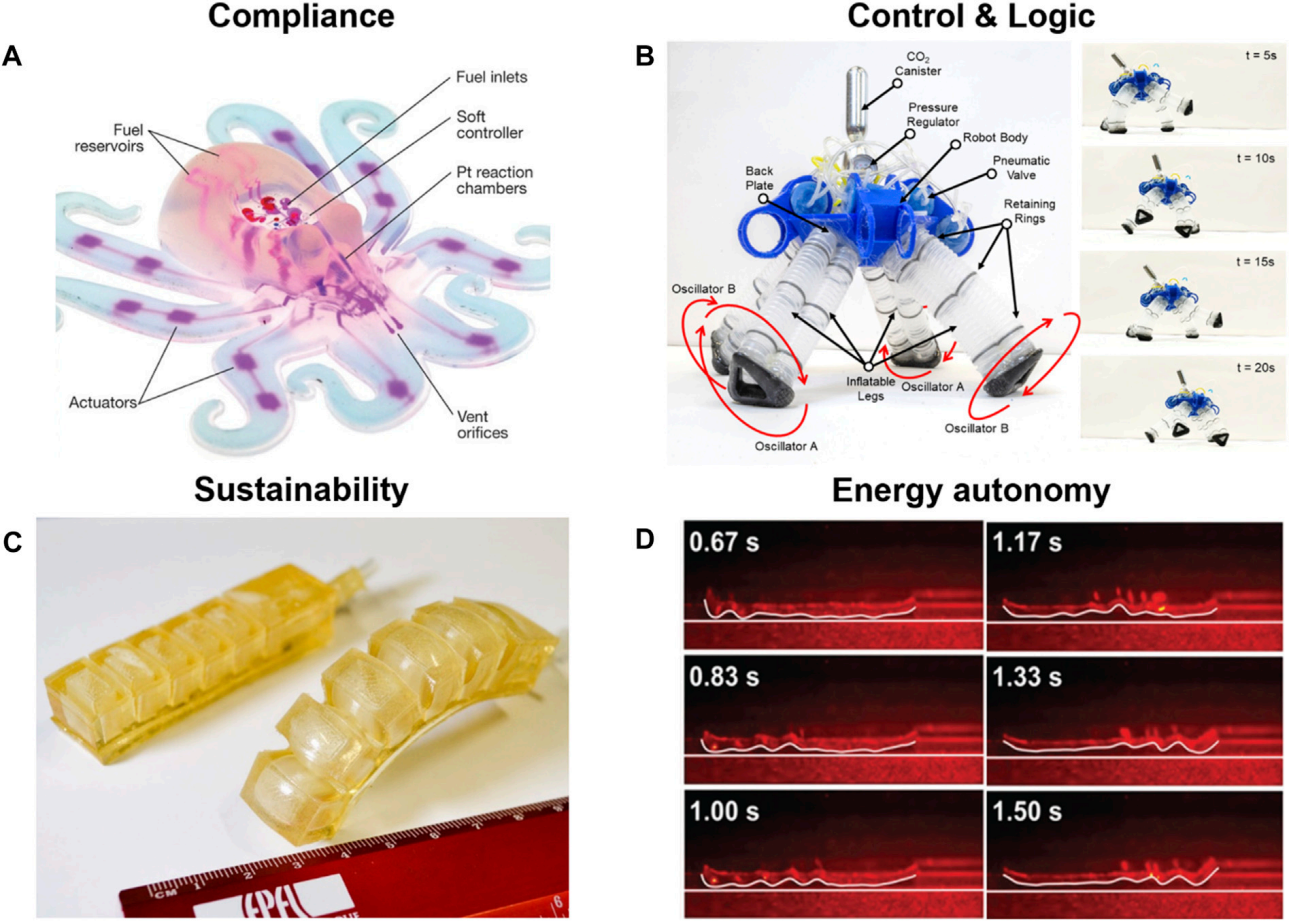
Soft robotic examples for electronic free control, energy autonomy, sustainability, and compliance. **(A)** As an example of electronic free control, the untethered quadruped utilizes soft pneumatic logic gates set up as oscillators to control movement and object avoidance. From (Drotmann et al., 2021). Reprinted with permission from AAAS. **(B)** Liquid crystalline elastomer-based crawling robot driven by a laser passing along the robots length. This is an example of energy autonomy as the actuation and motion are generated by structural changes inside the material in reaction to light exposure, and no further energy source is needed. From (Rogóż et al., 2016). Reprinted with permission from John Wiley and Sons. **(C)** The gelatin-based 3D printing materials are used as an example of biobased, biodegradable, and sustainable materials. From (Shintake et al., 2017). Reprinted with permission from IEEE Proceedings. **(D)** The fully autonomous and fully flexible iconic Octobot is the world’s first fully flexible soft robot capable of autonomous movement. From (Wehner et al., 2016). Reprinted with permission from Springer Nature.

Seventy years ago, McKibben presented the first compliant actuators (Agerholm and Lord, 1961; Schulte, 1961), which marked the beginning of soft robotics (Whitesides, 2018; Esser et al., 2020). From there, inflatable compliant actuators (Baldur and Blach, 1985; Suzumori et al., 1991) were used as elements in continuum robots (Robinson and Davies, 1999), paving the path for fully flexible robots, such as the multigait soft robot (Shepherd et al., 2011) or the soft autonomous Octobot (Wehner et al., 2016) (Figure 1A). With the advent of soft actuators and soft robots, the field of bio-inspired robotics evolved to the point that recently a bio-inspired robot autonomously floated on the bottom of the Mariana trench, raising the bar from simply compliant to fully flexible autonomous soft machines (Li G. et al., 2021).

Adaptivity, variability, continuity, and basically infinite degrees of freedom (DoFs) characterize current bio-inspired soft robots (Pfeifer et al., 2012; Coyle et al., 2018; Li et al., 2019; Mazzolai and Laschi, 2020; Zeng et al., 2020; Ilami et al., 2021). Such systems strive to be autonomous but also compliant like their natural counterparts (Sadeghi et al., 2017; Chubb et al., 2019; Mazzolai and Laschi, 2020; Bai and Shepherd, 2021; Mazzolai et al., 2022). However, for complex autonomous motions, an electronic controller together with an on-board battery are usually required, which compromises and even contradicts the idea of compliant soft systems (Bledt et al., 2018; Katz et al., 2019; Xu and Perez-Arancibia, 2020; Drotman et al., 2021; Decker et al., 2022). This highlights the problematic contradictions in soft and bio-inspired robotics. Systems need to be simple, autonomous, compliant and self-sustained to move, collect data and interact with the environment, but to achieve this goal, we build complex electronically controlled heavy systems.

This perspective paper highlights and discusses current contradictions in the field from a young researcher’s point of view. Through the discourse of two young scientists, current contradictions in the field of fully flexible autonomous systems are presented. In this context, the need for electronic controls, on-board energy supply, sustainability, and other relevant issues for bio-inspired soft robots are discussed and possible solutions are highlighted, leaving outlined perspectives open to the reader’s personal interpretation. In conclusion, a new promising but slightly obscured application area for bioinspired soft robotics is highlighted.

## 2 Are the soft robots really better than the robots that we have now?

Soft robots are compliant machines often fabricated from flexible materials such as silicones or thermoplasts. Recent developments enable their manufacturing *via* 3D printing using soft inks or flexible TPU filaments (Wehner et al., 2016; Slesarenko et al., 2018; Skylar-Scott et al., 2019; Conrad et al., 2020; Conrad et al. 2021; Conrad et al. 2022; Kappel et al., 2022). Most soft robots utilize compliant pneumatic actuators from flexible materials for movement (Whitesides, 2018; Esser et al., 2019a). The pneumatic actuators are mostly outfitted with a strain-limiting layer or thicker walls on one side, creating non-symmetrical conditions leading to directional bending. Based on these bending motions, many different soft robotic systems were developed, including bioinspired grippers, crawlers and even walkers (Shepherd et al., 2011; Whitesides, 2018; Xu and Perez-Arancibia, 2020; Drotman et al., 2021; Hubbard et al., 2021; Decker et al., 2022) (see Figure 1).

Soft robots are not only ideal for research in harsh environments, as the system could be crushed and still work afterward (Seok et al., 2013), but are also being integrated into industrial production lines and healthcare. These systems are ideal for robot-organism and human-robot interactions because the “interacting” parts of the systems (actuators and grippers) are mostly soft and compliant. Through flexible grippers, flexible suction cups and even completely compliant robot arms, the risk of injury in an integrated production line can be reduced tremendously (Grzesiak et al., 2011; Whitesides, 2018). Compliant systems can be equipped with a variety of sensors for system monitoring, object perception or interaction with their environment. Their characteristics enable bio-inspired soft robots to work in extreme and harsh environments in which electronic systems normally fail, like deep sea high-pressure regions (Mariana trench) (Li G. et al., 2021), radiation-contaminated or exposed areas (like Chernobyl, Fukushima and nuclear reactors) (Yirmibeşoğlu et al., 2019), mining or in space applications—in which electronics add weight, raise system complexity and costs.

The concept of soft continuously-deformable robots is very intriguing, however, it faces some crucial obstacles that are hard or might be physically impossible to overcome. Robots, in general, are required to perform some action interacting with the environment, such as moving around or grasping other objects. However, the generation of force large enough to hold objects or overcome gravitational force becomes much harder with an increase in the size of the soft robot body. Soft robot bodies cannot be too compliant, however, stiffer robots usually require more energy to deform. For example, in order to hold 0.6 kg, a precharged pneumatic bending actuator with a total length of 14 cm requires approximately 1 bar of pressure (Li Y. et al., 2018). Partially due to poor scalability, soft robotic systems often utilize miniature (micro and millimeter-sized) components, such as cilia (Rockenbach et al., 2015; Zhang et al., 2018; Milana et al., 2020). It mostly holds true also for soft robots that employ stimuli-responsive materials (Medina-Sánchez et al., 2018; Tyagi et al., 2021). Medium-sized soft robots usually have to play another trick to escape gravity by functioning underwater, as gravity constantly tries to deform the compliant body (Aracri et al., 2021). In general, the balance between the body’s compliance and its ability to perform any meaningful task at the same length scale is hard, and large soft robots are not viable compared to their classical counterparts. With this in mind, what is the reason to care about safe human-robot interaction if soft robots will never be large enough to harm humans?

## 3 Are electronic-free soft robots feasible and useful?

It is easy to see that electronic components make soft robots more bulky and definitely compromise compliance. As shown by Octobot (Wehner et al., 2016) (Figure 1A), it is technically possible to perform sensing and very simple decision-making without any electronic components. Applying the principles of analog operators in electronic circuits and fluidic logic, novel electronic-free soft logic gate systems can be developed. Pneumatic pressure drives these systems capable of rudimentary computational tasks. Current research focus lays on using basic logic gates like AND, NOT, OR, XOR in soft robotic systems (Preston et al., 2019a; Preston et al., 2019b; Mahon et al., 2019; Nemitz et al., 2020; Xu and Perez-Arancibia, 2020; Drotman et al., 2021; Hoang et al., 2021; Hubbard et al., 2021; Pal et al., 2021; Rajappan et al., 2021; Decker et al., 2022; Kendre et al., 2022; van Laake et al., 2022) (Figure 1B). Another important function of electronics in robotics is to provide the ability to sense. In soft robotics, sensing might be realized through the use of smart stimuli-responsive non-electronic materials, such as shape memory alloys (SMAs) (Kim et al., 2014; Rodrigue et al., 2017), shape memory polymers (SMPs) (Lendlein, 2018), hydrogels (Lee et al., 2020; Skarsetz et al., 2022), or liquid crystal elastomers (LCEs) (Rogóż et al., 2016; Kularatne et al., 2017; Lall and Zappe, 2022) (Figure 1D). The large family of flexible sensors that can be integrated into soft robotic systems is based on stretchable conductive materials, such as carbon nanotube/polyurethane fibers (He et al., 2019; Kar et al., 2022), conductive polymers (Fan et al., 2019) or conductive hydrogels (Shen et al., 2022). Among them, ionic flexible sensors (IFS) are one of the most interesting for bio-inspired design since, similar to living organisms, they use ion migration to sense external stimuli (Amoli et al., 2019; Zhao et al., 2022), which makes them attractive for potential applications in artificial skin or artificial organs. In general, ongoing research in soft materials that aims to improve their mechanical properties (Zhao, 2017) or grant them new functionalities (Herbert et al., 2018; Bartlett et al., 2019; Khatib et al., 2021) can be quite easily picked up by the field of soft robotics (Terryn et al., 2017). However, the great challenge lies in combining these scientific and engineering achievements in functional and easy-to-produce soft systems. One focus area of soft robotics dedicated to such systems is bio-inspired plant robotics, with the example of artificial Venus flytrap systems (AVFs) (Esser et al., 2020; Tauber et al., 2022). AVFs try to mimic the functions of a real plant by reacting to “prey,” sensing environmental conditions and harvesting energy from the environment. AVFs often use actuators that react to a change in temperature (SMAs, SMPs), humidity (hydrogels) or light (LCEs) with a closure of the trap lobes (Esser et al., 2020). Integration of energy harvesting structures like flexible solar cells (Pagliaro et al., 2008; Roldán-Carmona et al., 2014; Jung et al., 2019; Zimmermann and Würfel, 2020; Horii et al., 2022), solar batteries (Büttner et al., 2022) or solar supercapacitors (Berestok et al., 2021; Berestok et al., 2022; Delgado Andrés et al., 2022) might provide enough energy to supply sensors or low energy actuators in the future; for more details on energy harvesters, please look in Section 5. With such systems, autonomous bio-inspired or soft robots could be powered and outfitted to gather sensory data, e.g., in as small scale UAVs or drones (Han et al., 2009; Jafferis et al., 2019), or to interact with the users and environment as autonomous fully flexible healthcare robots or devices.

As already mentioned, any robot has to perform meaningful tasks, therefore, it requires a control system that is either preprogrammed or can make a decision depending on the available information. Our silicone-based chips are amazing at doing exactly that! This is why while heavy and powerful motors of well-infamous Atlas (Guizzo, 2019; Nelson et al., 2019) provide it with enough force to make a backflip, a very small and energy-efficient silicone chip performs all processing and computational heavy-lifting. While successful attempts to implement basic elements of electronic circuits using pressure instead of electric current were reported, such electronic-free processing units are in their infancy and can be compared with the Antikythera mechanism from 100 BC (Freeth et al., 2006). Similar to how mechanical computing hit a wall before the invention of electro-mechanical (and further fully electrical) computing (Williams, 1985), it is hard to imagine that “soft” computing will be capable of even simple decision-making without electronic components. At the same time, it would be wrong to completely ignore the recent renaissance of mechanical computing (Yasuda et al., 2021) that, in contrast to previous realizations, started to utilize the compliance of materials. However, while the manufacturing techniques continue to be further developed and now technically allow the fabrication of sensitive elements and membranes with micrometer precision (Hines et al., 2017), the issue is in the stability of the system and its capacity to survive over thousands and millions of cycles without failing regardless of actuation method [pneumatic (Shepherd et al., 2011; Whitesides, 2018), temperature (Lendlein and Kelch, 2002; Lagoudas, 2008), light (Lim et al., 2017; Wani et al., 2018)]. Surprisingly, this very important aspect is often omitted in research papers. Stability is especially crucial for non-volatile memory, which is necessary for the reprogrammability of soft (pneumatic) circuits. Regardless of the methods of memory storage [usually *via* bistable beams or membranes (Nemitz et al., 2020; Chen et al., 2021)], a smaller size means a higher probability of accidental switch due to the overall deformation of the soft body or simple leak, which is a solved problem in the case of Si-based electronics.

Finally, referencing the most famous biological inspiration for soft robotics, we recall that if an octopus can fit its beak through a hole, it can squeeze through it completely. If the soft robot needs to work in a complex environment (e.g., nuclear reactor), it has to have a non-soft camera to evaluate the situation and provide feedback. The addition of miniature electronic components (preferably redundant and distributed) will not undermine its compliance while drastically improving its performance. Therefore, fully electronic-free autonomous soft robots will be used only for relatively simple tasks that require repetition of a set of preprogrammed actions [e.g., heart beating (Rajagopal and Hoeper, 2016)]. For more universal applications, a synergy between soft components and classical electronic schemes has to be maintained. Achieving a paradigm shift with soft robotic systems would necessitate not only the combination of soft and hard electronic components but also the use of novel materials, as soft robots are not reliable enough so far.

## 4 Are soft robots sustainable in the circular economy of the future?

Circular economics and sustainable living are the cornerstones of the recently adopted government policies in the European Union (EU Commission, 2020) and the US (US Environmental Protection Agency, 2021). With this, the notion of bio-inspiration in soft robotics should be expanded to mimic the lifecycle of biological organisms. At first sight, it might appear that the absence of electronic systems in flexible soft robots will be beneficial from a sustainability point of view. Indeed, electronic components are known to be hard to recycle, and 57.4 million tons of electronic waste (e-waste) were generated in 2021 alone. However, this statistic might be a bit misleading in relation to our topic since small electronic components can be reused and repaired, which is one of the points of a UN report devoted to e-waste (UN Environmental Programme, 2019). Moreover, after simple reprogramming, the same electronic component can perform different routines, removing the need to fabricate an absolutely new soft robotic body for each new task. Due to extremely large design space and lack of the generally accepted standardized framework for soft robots, their reuse is non-existing. Therefore, soft robots can rely only on recycling rather than on reuse, while the latter is a superior option from the waste management hierarchy (UN Environmental Programme, 2013). While some of the very popular soft materials in robotics [e.g., silicone rubber Ecoflex (Siegenthaler et al., 2012)] are biodegradable, others (e.g., acrylate-based photopolymer resins for 3D printing) can be toxic (Zhu et al., 2015) and not recyclable (van Bochove and Grijpma, 2019). Therefore, the development of new compliant materials that will satisfy sustainability criteria will often be necessary (Maines et al., 2021; Zhu et al., 2023). However, even after a thoughtful assessment of specific material sustainability, there is a risk that sustainability factors might hinder progress in the field, especially if more strict environmental policies will be adopted in the future, replacing and updating the existing ones (EU Parliament, 2006).

If the requirements for sustainability are kept in mind while designing bioinspired soft robots, one can already achieve a partially circular economic use of soft robots. A successful recycling concept requires that the individual robot materials can be easily separated to enable uncomplicated reuse, replacement and upgrading of robots (Hartmann et al., 2021). This could be achieved if we use already existing easily recyclable sustainable options, such as biobased materials from renewable resources. Green composites could be used for parts that demand high structural integrity (Koronis et al., 2013; Calvino et al., 2020); protein-based materials (Huber et al., 2022), gelatin-glycerol hydrogels (Shintake et al., 2017) and DNA-based hydrogels (Walther, 2019; Akintayo et al., 2021) could be used as biodegradable and compostable actuators. Soft robots are suitable candidates for the use of such materials as they are often monomaterial systems with low material complexity. If bio-based soft and hard materials like polyhydroxyalkanoates (PHA), polylactic acid (PLA), poly(lactic-co-glycolic acid) (PLGA), gelatin gels and starch blends are used for the makeup of such systems, it will increase their recyclability and sustainability (Bagheri et al., 2017; Shintake et al., 2017; Hartmann et al., 2021) (Figure 1C). Current systems even highlight the use of sustainable materials for the electronics in soft robots, such as biodegradable sensors (Hartmann et al., 2021) based on PLLA nanofibers (Curry et al., 2020), cellulose nanofibers (Gao et al., 2019) or waxes doped with conductive particles (Won et al., 2018). However, some applications would still necessitate materials or composites that are not easily recyclable. For such systems, materials with features like self-healing or self-hardening for damage avoidance and longer life cycles would be beneficial (Terryn et al., 2017; Markvicka et al., 2018; Li et al., 2019; Meesorn et al., 2019; Terryn et al., 2020; Bai and Shepherd, 2021; Speck et al., 2022). Changing the systematic design into a compartmentalized modular design (Pfeifer et al., 2012; Pfeifer et al., 2007; McKenzie et al., 2018), in which parts can be interchanged or repurposed, would create even more sustainable soft robots. The use of not only bio-based and recyclable materials but also biocompatible and bioresorbable materials would enable novel application fields for bio-inspired soft robots in medicine (Hartmann et al., 2021). With the further advances in the industry of soft robotics, employment of more sustainable materials for soft bodies as well as standardization of the soft robot modules, will be necessary.

## 5 Can soft robots be useful without power packs?

From a physical point of view, to perform any work (crawl, grip, deform, etc.) robot might consume energy regardless if it is rigid or soft. Therefore, energy needs to be stored, delivered to the point of action, and conversed to mechanical motion or deformation. The necessity to supply energy drastically sophisticates robots’ autonomy. Traditional autonomous robots (e.g., vacuum cleaner robots) usually rely on electrochemical batteries (e.g., Li-ion), which after multiple breakthroughs (Reddy et al., 2020), reach an energy storage density of 700 Wh/L (or specific energy of 250 Wh/kg). Unfortunately, the field of soft robotics does not go along with such batteries. The main reason here is the bulkiness of batteries and other components, such as motors transforming electrical voltage into mechanical motion. If soft robots want comparable autonomy, other ways to store and transduce energy must be developed (Aubin et al., 2022). Here two principally different ways to supply energy for soft robots can be distinguished. The first approach relies on capturing energy from a purposefully created and controlled external field (usually magnetic). The robot can harvest its energy and move when placed inside such a field (Huang et al., 2015; Hu et al., 2018). This is up to debate if robots functioning in an external magnetic field can be considered “truly” autonomous. In general, this is a viable idea for microrobots, as shown, for instance, in the biomedical field (E. Peyer et al., 2013; Li J. et al., 2018), however, this approach cannot be scaled up. Therefore, larger soft robots have to rely on energy stored inside their soft bodies (Wehner et al., 2014). A soft pneumatic robot, for instance, can use stored compressed air or has the ability to generate pressure using a chemical reaction happening in the small reactor inside the soft body (Onal et al., 2017). However, from the point of energy density, compressed-air energy storage is inferior to electrochemical batteries, and this disadvantage amplifies manifold with a decrease in robot size. Similar conclusions can be made concerning other energy storage methods [e.g., *via* elastic energy (Pal et al., 2021)] that cannot really compete with well-established electrochemical batteries. A very good comparison of different ways to store and transform energy in relation to soft robotics was recently presented by Aubin et al. (2022), however, the usage of energy density and power density without accounting for the size (length scale) of the robot might be a little bit misleading. Considering the limited applicability of soft robots in general, it might be more beneficial to explore ways of integrating soft elements into already existing robotic systems rather than attempting to design fully autonomous soft robots working on new principles.

Equipping soft robot systems with energy-harvesting structures would make them primarily autonomous. Not all autonomous robots always need energy, but if they need it, the energy must still be sourced from storage. Here a distribution of batteries over the whole body near the consumers (actuators or sensors) would decrease the size and weight of the system. As mentioned above, with the use of sensors and actuators with low energy consumption, the need for powerful power supplies becomes less prominent. Current research focuses on self-powered sensors, and there are already sensory systems for soft robots solely driven by energy harvesters. For instance, Chen et al. (2020) integrated a triboelectric nanogenerator (TENG) into a pneumatic bending actuator, generating electricity by contact and separation while bending. Next to system integration, the makeup of harvesters itself can be a challenge as for the use in soft robotics, they need to be lightweight, robust and ideally based on flexible materials. Usable systems in this case are flexible solar cells (Pagliaro et al., 2008; Roldán-Carmona et al., 2014; Jung et al., 2019; Zimmermann and Würfel, 2020; Horii et al., 2022), solar batteries (Büttner et al., 2022) or solar supercapacitors (Berestok et al., 2021; Berestok et al., 2022; Delgado Andrés et al., 2022) as well as triboelectric nanogenerators (TENGs) (Wu et al., 2019; Wang, 2020), piezoelectric nanogenerators (PENGs) (Lee et al., 2018), thermoelectric generators (TEGs) (Sherkat et al., 2022), biofuel cells or microbial fuel cell (MFC) (Ieropoulos et al., 2005; Kim et al., 2007; Tauber and Fitzgerald, 2021) and hybrids system, e.g., of TENG and biofuels cells (Liu et al., 2022). These can generate electricity to power low-energy consumers for autonomous wearable sensing and transmit sensory data to microprocessors and computers. The problem here is the energy consumption of transmitter structures that ranges from a few milliwatts to hundreds of milliwatts (Liu et al., 2022). The output of a self-charging power source should reach at least tens of milliwatts to support a fully independent portable device (Liu et al., 2022). As these systems convert energy from the environment, harvesters should be designed to have access to energy sources such as heat, temperature gradients, light, body fluids, and so on. Triboelectrics and piezoelectrics harvest energy from motion; these could be used to harvest mechanical energy *in vivo*. MFCs can convert nutrition and bodily fluids into energy (Ieropoulos et al., 2005; Kim et al., 2007; Tauber and Fitzgerald, 2021), TENGs can convert heat into energy, making them ideal for application on the outside of a soft robot or in an inner fluid channel. Flexible solar cells, solar batteries and solar supercapacitors need—as the name already states—access to light. There are already a few fully integrated actuators, energy harvesters and sensing systems like the MXene-based soft actuators by Li et al. (2021b), but they are not useable for large systems due to very limited generated force. The above-mentioned examples highlight the benefits and possible applications of soft robots without power packs. Ideally, the energy requirements of the used sensors and actuators should not exceed the energy provided by the harvesters. If this should happen, decentralized flexible energy storage solutions can be used without hindering the compliance of the system.

## 6 The future of autonomous, sustainable bio-inspired soft robots as artificial organs

Summarizing previous observations, we see that soft robots can outperform their traditional counterparts, be sustainable, capable of harnessing energy, and simultaneously operate in severe settings. With the progress in the field of soft robotics, new concepts and ideas initiated in the academic environment can be then picked up by commercial enterprises for further development. By working towards functionalization, diversification and final commercialization, academy and engineering can provide broader adoption and acceptance of soft robotic systems. For example, throughout more than a decade of development, soft robotic grippers (Amend et al., 2016; Hughes et al., 2016; Shintake et al., 2018; Wang et al., 2022) have already achieved a mature technological readiness level (TRL) and can be employed in commercial and industrial applications (Soft Robotics Company, 2019; Negrello et al., 2020). Nevertheless, while further incremental progress in soft grippers is inevitable, existing technological applications [e.g., in the food industry (Wang et al., 2021)] do not unlock the full potential of soft robotics. Therefore, since the human body provides one of the harshest environments, additionally imposing very strict requirements on the biocompatibility of materials, we believe that one of the most perspective directions for soft robotics is associated with the design of artificial human organs. The necessity for such systems is not only highlighted in medical reports, papers and books (Malchesky, 2001; Miller, 2006; Rajagopal and Hoeper, 2016) but also acknowledged by the existence of a Horizon2020 EU project on the subject called “Development of the first fully biocompatible, soft actuated heart: combining *in situ* tissue engineering and soft robotics” (Horizon, 2022). There are still millions of people all over the world waiting for transplants and new organs. Currently (December 2022) in the US alone, 1,05,703 people are in need of an organ transplant (UNOS, 2022), and in 2020 ten patients on average died per day waiting for an organ transplant in Europe (Spanish National Transplant Organization, 2022).

Currently, patients in severe cases of organ failure are connected to extracorporeal artificial organ devices like heart-lung machines (in case of heart or lung failure) (Rajagopal and Hoeper, 2016) or hemodialyzers (in case of kidney failure) (Gaudry et al., 2022). A temporary solution for patients on the waiting list could be rudimentary organ prostheses. There are already a few systems, but these lack simplicity, are not electronic free and prone to failure. Some systems like the soft total artificial heart (Cohrs et al., 2017), the SoGut—artificial stomach peristalsis simulator (Dang et al., 2020), the artificial oesophagus system RoSE (Dirven et al., 2013; Dirven et al. 2015; Dirven et al. 2017) and the silicon-based peristaltic pump (utilizable as an artificial intestine and oesophagus) (Esser et al., 2019a; Esser et al., 2019b; Tauber et al., 2021; Tauber and Fitzgerald, 2021) are completely soft but necessitate an external pressure supply (Menciassi and Iacovacci, 2020; Tauber and Fitzgerald, 2021; Hashem et al., 2022). There are a few commercially available artificial hearts, such as the temporary Total Artificial heart (TAH-t) (SynCardia Systems, Inc., Tucson, AZ, United States) and the AESON CARMAT TAH (Carmat, Vélizy-Villacoublay, France). The TAH-t from SynCardia is approved by the US FDA (Food and Drug Administration) and got a CE mark in Europe as a “bridge to transplantation” therapy device (Cohrs et al., 2017). In 2021 there was an Urgent Field Safety Notice (Reference 10741/21) from the German Federal Institute for Drugs and Medical Devices (BfArM) describing a problem with a CPC connector to result in patient harm. Although these are just for the CPC connectors that fasten the TAH-t cannulae to the external driveline, this could still cause harm to a patient and highlight the complexity of such systems and the necessity for simpler (easy to use) systems. Bioinspired soft robotic systems show usability as artificial hearts as these can recreate the pulsatile flow of the human heart using squid and jellyfish like pulsatile motion (Roche et al., 2017; Bujard et al., 2021).

**FIGURE 2:**
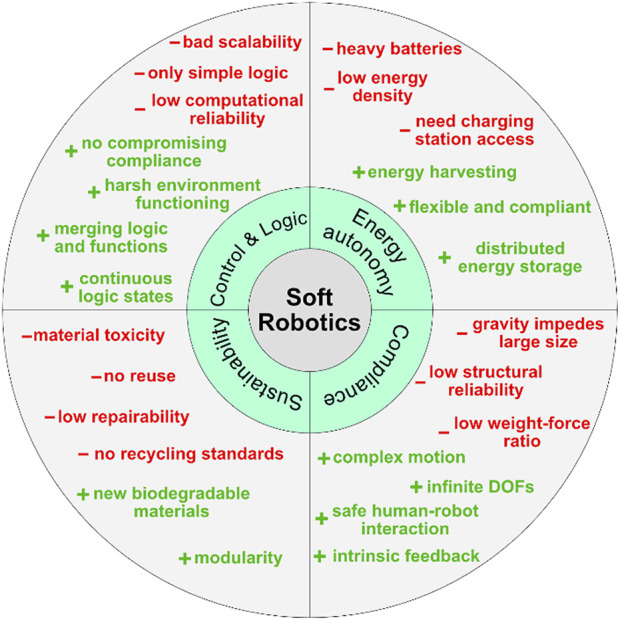
Selected positive and negative aspects of soft robotic systems.

If the above-described goals for bio-inspired systems are met, we would create the building blocks for autonomous, electronics-free, sustainable, biocompatible systems, such as artificial organs. We have identified the need for such systems, the areas in which current systems are deficient, and how the technologies proposed here could address these shortcomings (Figure 2). First rudimentary systems highlight that the actuation technology to create biologically feasible motions is already there but only needs to be made autonomous and manufactured from sustainable biocompatible materials. Rapid prototyping and novel fast medical imaging technologies (CT, fMRI, X-Ray) could generate prostheses specifically adapted to the patients’ physique and needs. With the approach described above, we want to incentivize achieving a goal that will help millions of people.

## Data Availability

The original contributions presented in the study are included in the article/supplementary material, further inquiries can be directed to the corresponding authors.
